# Quality of Life and Performing Acupuncture on 150 Patients Suffering From Chronic Pains: A Randomized and Intervention Study Before and After Performing

**DOI:** 10.5812/ircmj.1411

**Published:** 2013-03-05

**Authors:** Tahereh Fathi Najafi, Mahshid Hejazi, Mojtaba Meshkat, Soheila Hajibabakashani

**Affiliations:** 1 Department of Midwifery in Medical University, Mashhad Branch, Islamic Azad University, Mashhad, IR Iran; 2 Department of Foreign Languages, Mashhad Branch, Islamic Azad University, Mashhad, IR Iran

**Keywords:** Acupuncture, Quality of Life, Pain

Dear Editor,

Pain is the commonest complain of human being. If it becomes chronic it affects all the dimensions of life quality. There are many ways to relief pain but some of them have disadvantages that make not well feeling for patients. Currently, people have been attracted to complementary treatments and one of the main ways to cure chronic pain is acupuncture (1-4). Two extensive studies in Germany have shown the effects of acupuncture on the life quality of the patients suffering from chronic diseases 1.According to WHO the best definition of quality of life is: “understanding one’s situation in life based on the culture, the evaluative system with which one is living, the goals, expectations, standards, and priorities (5). The purpose of this study is to examine the effect of acupuncture on the quality of life on 150 patients who suffered from chronic pain. This study is a descriptive and intervention type before and after of intervention. All patients were suffering from arthritis pain, migraine backaches, muscular spasms, pain in the neck, and in the knee. The patients went through acupuncture for 8 -10 sessions and either once or two times a week. The number and the place of needles were 8 -12 for each patient which were located on the right place and acupuncture was done by Dr Sadr Nabavi who has a specialist in acupuncture. Data analysis was done by SPSS software. T –test and variance analysis was done at the meaningful level of 5%.The life quality questionnaire (WHOQOL) of the World Health Organization included have 4 domains. 77.3% i.e. of the participants were women. 22.7% i.e. were men. The highest age range were between 41 -50 (28%) and the lowest were between 71 -75 (4.7%).46% of the pain was related to backaches, rheumatoid arthritis 16.7%, migraine 14.7% and the smallest rate goes to other disorders like pain in the neck 9.3%.). After 3 months of acupuncture, it was revealed that Life quality is changed (P = 0.0001 and P = 0.0001 respectively) in Environmental and Physical domains in women. All domains in quality of life don’t show a significant difference in men (Table 1). The rate of Physical domain increases in the patients who are over 60 years old and under 31 i.e. (P = 0.004 vs. P = 0.174). Psychological domain doesn’t difference before and after acupuncture in different age groups. (P = 0.2 to P = 0.8 for all age groups) (Table 1).

**Table 1.** Quality of Life Before and After Acupuncture Based on Gender

Variant	Age, y (Less)	Age, y (Most)	Age, y, Mean ± SD	P value
**Male**				
**Physical**				0.068
Before	18	68	52.3 ± 10.9	
After	39	75	54.9 ± 8.5	
**Psychological**				0.055
Before	25	75	53.1 ± 10.2	
After	29	71	50.6 ± 7.5	
**Social**				0.915
Before	0	100	56.6 ± 25.9	
After	17	92	56.9 ± 17.7	
**Environmental**				0.091
Before	31	91	59.7 ± 15.6	
After	38	91	62.1 ± 13.0	0.525
**Total**				
Before	31	80	55.4 ± 12.2	
After	38	70	56.1 ± 8.3	
**Female**				
**Physical**				0.001
Before	7	82	51.5 ± 15.6	
After	11	82	54.4 ± 10.9	
**Psychological**				0.737
Before	25	75	52.4 ± 11.7	
After	33	75	52.2 ± 8.6	
**Social**				0.063
Before	0	100	57.0 ± 25.4	
After	17	100	59.7 ± 21.2	
**Environmental**				0.0001
Before	22	97	60.9 ± 17.3	
After	22	97	63.8 ± 15.2	
**Total**				0.002
Before	20	89	55.4 ± 14.0	
After	30	88	57.5 ± 11.1	

Our study revealed that the Physical and Environmental domains of women suffering from muscular –skeletal diseases are better than before acupuncturing. (respectively P = 0.0001, P = 0.0001), while psychological and social doesn’t show it. (respectively P = 0.083, P = 0.208).”Wang, Hunter and Hoseiabadi and their colleagues” showed a meaningful difference in Physical and Environmental domains (6-8). In this study the best life quality improvement in Physical domain has been seen in women. Uthaikhup and Plank also confirm our findings (9, 10). All domains don’t show difference in before and after acupuncture in men it may be depend on their attitude to complementary medicine in Iran. However, acupuncture can be considered as a useful way of treatment for the patients who suffer from chronic muscular- skeletal diseases.
